# Effects of Exposure to Semiconductor Nanoparticles on Aquatic Organisms

**DOI:** 10.1155/2012/397657

**Published:** 2011-10-27

**Authors:** Kenton Leigh, Jennifer Bouldin, Roger Buchanan

**Affiliations:** ^1^Graduate Program in Molecular Biosciences, Arkansas State University, P.O. Box 837, State University, Jonesboro, AR 72467, USA; ^2^Ecotoxicology Research Facility, Arkansas State University, P.O. Box 847, State University, Jonesboro, AR 72467, USA

## Abstract

Because of their unique physical, optical, and mechanical properties, nanomaterials hold great promise in improving on a wide variety of current technologies. Consequently, their use in research and consumer products is increasing rapidly, and contamination of the environment with various nanomaterials seems inevitable. Because surface waters receive pollutants and contaminants from many sources including nanoparticles and act as reservoirs and conduits for many environmental contaminants, understanding the potential impacts of nanoparticles on the organisms within these environments is critical to evaluating their potential toxicity. While there is much to be learned about interactions between nanomaterials and aquatic systems, there have been a number of recent reports of interactions of quantum dots (QDs) with aquatic environments and aquatic organisms. This review is focused on providing a summary of recent work investigating the impacts of quantum dots on aquatic organisms.

## 1. Introduction

In 2006, the net global market value of nanotechnology was estimated to be US $10.5 billion [1] and is projected to increase to US $26.7 billion by 2015 [2]. As the use of nanomaterials increases, it seems inevitable that nanoparticles will be released into the environment. Despite the rapid increase in the use of nanomaterials in research, industrial, and consumer products, information about their possible effects on organisms and the environment is lacking. Fortunately, there have been a number of recent reports relevant to understanding the fate and effects of nanoparticles in the environment. Many of these reports specifically address questions about the potential impacts of nanoparticles on aquatic environments. Because surface waters receive pollutants and contaminants, including nanoparticles, from many sources and act as reservoirs and conduits for many environmental contaminants, understanding the potential impacts of nanoparticles on the organisms within these environments is critical to evaluating their potential toxicity. At present, there is little information about how many nanoparticles enter aquatic environments and their routes of entry, but potential routes include atmospheric deposition, leaching from soil, direct input from wastewater discharges [3], and ground water reservoirs [4]. Evidence for possible contamination of water sources with nanoparticles has already been reported by Mueller and Nowack [5], who developed a model and estimated that levels of silver and TiO_2_ nanoparticles and carbon nanotubes in freshwaters at the time were 0.3, 0.7, and 0.0005 *μ*g L^−1^, respectively. While, as shown by the reports reviewed herein, knowledge of nanomaterial interactions with aquatic environments is increasing, there is still much that must be learned. This review is focused on providing a summary of recent work investigating the impacts of quantum dots (QDs) on aquatic organisms. 

Quantum dots (QDs) are a class of nanoparticles that, because of their unique optical properties, show great potential for incorporation into numerous consumer, industrial, and medical products. QDs are semiconductor fluorescent nanocrystals whose unique chemical, physical, and optical properties make them ideal for use in a variety of applications in electronics, computing, and many biomedical applications. Current and projected uses include LED displays and lighting [6], biomedical imaging and probes [7, 8], security inks [9], quantum computing applications [10], photovoltaics [11], and photodynamic cancer therapies [12]. 

While QDs have many unique characteristics, one of their most important features is their fluorescence. Because the fluorescence properties of the particle are inherent in the structure and composition of the metallic core, they are much brighter and more stable than most chemical fluorophores. Most commercially available QDs are multilayered, nearly spherical particles with a metallic core surrounded by a series of protective shells. The core of a QD is a heterogeneous metallic nanocrystal composed of semiconducting metals. Although there is considerable ongoing investment in producing QDs with less toxic metals, at present, most commercially available QDs have a nanocrystalline core of cadmium selenium (CdSe) or cadmium tellurium (CdTe). Unfortunately, these are known to have adverse effects on organisms and the environment [13–15]. This core is often encased in another semiconductor shell that helps maintain structural integrity and enhances and stabilizes its mechanical and fluorescent properties. This layer is typically made of ZnS. The diameter of the resulting structure ranges from 2 to 10 nm. Fortunately, the ZnS shell seems to at least temporarily prevent the leaching of toxic metals from the core. In most cases, commercial QDs are then coated with a hydrophilic coating (e.g., polyethylene glycol). This usually renders the particles miscible in water, a property that is critical for many applications (e.g., biomedical imaging). The outer surface of the polymeric coating can be treated so that active –COOH or –NH_2_ groups are available. When altered, the surface of a single QD contains 10–100 possible surface attachment groups which can be conjugated to biomolecules such as peptides, antibodies, and oligonucleotides. The final diameter of the functionalized QD can range from 10 to 100 nm. 

Although commercial QDs are available from several sources, many investigators use laboratory-produced QDs. QDs are relatively easy to make, so producing them in the laboratory reduces the cost of experiments and allows custom-designed QDs with coatings not readily available from commercial sources. Laboratory-manufactured QD cores, without coatings or shells, have also been used in a number of studies. However, careful purification and characterization of laboratory-made QDs is critical to accurate interpretation of experimental results. Laboratory-made QDs also tend to have different aggregation properties from commercially available QDs. If these properties are well characterized, this can be a significant advantage because the effects of aggregated QDs can be much different than those of nonaggregated QDs, allowing these differences to be characterized. Because the effects of exposure to aggregated QDs often differ from exposure to individual particles, aggregation tendencies have the potential to greatly impact effects of QDs on aquatic environments. The type of outer coating on these QDs also has profound effects on the results of exposure. In some cases, the coating material is itself toxic; therefore, QDs coated with these materials exert toxic effects resulting from the coating rather than the QDs or the metals they contain. Several reports discussed below used laboratory-made QDs, tested the toxicity of the coating material, and show this effect. However, the toxicity of the coating is not always evaluated, so in some cases, it cannot be ascertained if the reported effects of exposure are the result of exposure to the coating material, to the QDs themselves, or both.

The impact of QDs on aquatic environments is of special concern because in many commercially available formulations, they, unlike many carbon-based nanoparticles, are completely miscible in water. This property renders QDs especially susceptible to structural alterations and degradation as they interact with biotic and abiotic environmental factors. Because many QDs contain metals, structural alterations may result in the release of metals into aquatic systems. Although the impacts of many metals on aquatic systems have been well studied, information about QDs' environmental fate and potential impacts on aquatic environments remains limited [16, 17]. Here, we specifically review recent reports about the effects of QD exposure on aquatic organisms.

## 2. Physiochemical Properties of QDs and the Aquatic Environment

Throughout this review, QD structure will be annotated as ^surface  ligands/coating^Core/Shell QDs. For example, a ^TOPO^CdSe/ZnS QD has a core of CdSe encased in a ZnS shell that is, in turn, coated with the nonfunctionalized organic ligand trioctylphosphine oxide (TOPO) and a ^COOH-PEG^CdSe/ZnS QD an identical core/shell structure, but is coated with polyethylene glycol (PEG) functionalized with carboxyl groups. Because the outermost surface of the QD interacts directly with biotic and abiotic environmental factors, the physicochemical properties of this surface are important determinants of the QD's environmental effects. The outer surface of the nanoparticle is also subject to degradation by biotic and abiotic environmental factors. This degradation may alter the surface chemistry and, more importantly, result in the release of the toxic metals in the QD core into aquatic environments. Thus, careful characterization of the QD surface is important because all QDs will not behave the same under similar environmental conditions, and it will ultimately determine the fate and effect on aquatic organisms. Therefore, we will investigate the literature describing how the various configurations interact with environment conditions to illustrate the importance of QD surface ligands on their behavior in aquatic environments.

### 2.1. Effects of QD Exposure on Algae and Microbes

Slaveykova and Startchev [18] studied the effects of natural organic matter and the freshwater green microalgae, *Chlorella kesslerii*, on the stability of carboxyl-polyethylene-glycol-(PEG-) coated CdSe/ZnS QDs. Fluorescence correlation spectroscopy was used to measure the physical effects that aquatic conditions had on QDs. At pH range of 5.0–8.0, there was no change in the number of fluorescent particles or on the fluorescence of each individual particle (as measured by count per particle, CPP) over 60 min. However, at pH of 4.0, the CPP of the particles significantly decreased. The authors suggest the phenomenon was possibly due to changes in the electronic environment of the ^PEG^CdSe/ZnS QDs and/or degradation of the QD structure. ICP-MS combined with microcon centrifugal filter devices (MCFD) showed that Zn^2+^ was present in the suspension, suggestive of QD degradation. The authors next tested the effects of ionic strength on the ^PEG^CdSe/ZnS QDs and reported that there was no significant effect on the number, average hydrodynamic radius (R_H_), and CPP of ^PEG^CdSe/ZnS QDs when the ionic strength of the suspension was between 0.1 mM and 10 mM, suggesting no aggregation or degradation under these conditions. Furthermore, at a pH of 7.0 with 5 or 15 mg  C L^−1^ (carbon per liter) of humic acid (HA) or 50 mg C L^−1^, extracellular polymeric substances (EPSs) had no measureable influence on ^PEG^CdSe/ZnS QD R_H_, CPP, or particle number. However, when 5 or 50 mg C L^−1^ alginic acid (AA) was added to a 20 nM ^PEG^CdSe/ZnS QD suspension at an ionic strength of 10 mM, the R_H_ of the ^PEG^CdSe/ZnS QDs was doubled, and there was a decrease in the fluorescence of individual ^PEG^CdSe/ZnS QDs. Therefore, the authors suggest that under these conditions, alginate could promote aggregation by acting as a “bridge” between individual ^PEG^CdSe/ZnS QDs by increasing the charge screen between QDs and alginate, thereby reducing repulsion between ^PEG^CdSe/ZnS QDs. When the microalgae *C. kesslerii* was added to a ^PEG^CdSe/ZnS QD suspension, there was no change in particle R_H_, but when 10^4^ g mL^−1^ cells were added to ^PEG^CdSe/ZnS QD suspensions, there was a decrease in the number of particles in the suspensions, suggesting that the algae enhanced ^PEG^CdSe/ZnS QD aggregation. Under these conditions, CPP was decreased, an effect that was also attributed to interactions between the ^PEG^CdSe/ZnS QDs and the algae. Overall, the algae had no measureable effect on the size of individual ^PEG^CdSe/ZnS QDs, but caused significant decreases in the fluorescence of individual QDs at their emission wavelength of 488 nm. The authors hypothesized this was due to changes in the suspension electronic environment, surface chemistry, or degradation of ^PEG^CdSe/ZnS QD as they interacted with the algae. Using ICP-MS, the percentage of Zn^2+^ in MCFD filtrates after 60 min of ^PEG^CdSe/ZnS QD/algae contact was 60%, compared with 37% in QD suspension that did not contain algae. However, the amount of Cd^2+^ MCFD filtrates was only 1% and 2%, respectively. Additionally, no blue shift in the emission of the ^PEG^CdSe/ZnS QDs was observed before and after contact with algae indicating that although there was degradation of the ZnS shell, no significant degradation of the core occurred during a 60 min exposure.

Wang et al. [19] investigated the effects that thioglycolate-capped CdTe QDs had on the growth of the unicellular green algae, *Chlamydomonas reinhardtii*. Before ^THIO^CdTe QD exposure, the authors measured the average particle size over a period of 48 h in algal culture medium. Interestingly, only trace amounts of nanoscale particles were observed after 24 h, while ^THIO^CdTe QDs aggregates of up to 710 nm were detected after 48 h. The authors attribute the aggregation to the high ionic strength of the culture medium which reduced the electric repulsion between particles allowing aggregation [20]. Therefore, it is important to note that the exposures in these experiments are most likely attributed to ^THIO^CdTe QD aggregates, and not nanoscale particles. Growth inhibition of *C. reinhardtii* cells was reported in cultures containing 1 mg L^−1^ or higher ^THIO^CdTe QD concentrations. When algae were exposed to 10 mg L^−1^ ^THIO^CdTe QDs, severe detrimental effects on cell growth were observed. ^THIO^CdTe QDs also induced cell aggregation in a dose-dependent manner. ^THIO^CdTe QD effects on the transcription of stress response genes encoding for superoxide dismutase (*sod1*) and glutathione peroxidase (*gpx*) were measured using RT-PCR. After a 3 h exposure to 0.1 mg L^−1^ QDs, a significant decrease in the levels of *sod1 *and a significant increase of *gpx* mRNAs were observed, and lipid peroxidation was increased. Significant increases in antioxidant genes were detected after exposure to 1 mg L^−1^ ^THIO^CdTe QDs, resulting in a significant increase of *cat* mRNA. 

Lin et al. [21] studied the effects that adsorbed CdSe/ZnS QDs capped with mercaptoundecanoic acid (MUA) ligands have on the algal species *Chlamydomonas*. The average hydrodynamic radius of the QDs was 11.69 nm, and ^MUA^CdSe/ZnS QD emission was 570–585 nm. Using bright-field microscopy, the authors observed that ^MUA^CdSe/ZnS QD-exposed cells were less mobile than control cells. UV-vis spectrophotometry showed that adsorption of  ^MUA^CdSe/ZnS QDs by algal cells increased in an exposure-dose-dependent manner. The concentration of adsorbed ^MUA^CdSe/ZnS QDs was shown to logarithmically increase up to ~0.05 ppm along with increased equilibrium concentrations of the ^MUA^CdSe/ZnS Ds (up to ~0.22 ppm). It was suggested that the affinity between the carboxylic groups of the surface ligands and the amine groups of the algal cell wall could be responsible for the adsorption. However, no ^MUA^CdSe/ZnS QD uptake into the algal cells was observed using electron microscopy, possibly due to ^MUA^CdSe/ZnS QD aggregation. The cell wall pore diameter was ~5–20 nm, so aggregates larger than that would not be able to enter the cells. However, ^MUA^CdSe/ZnS QD aggregation was not measured in these experiments. The authors reported decreased photosynthetic activity by measuring CO_2_ and O_2_ levels in the aqueous suspension after exposure to ^MUA^CdSe/ZnS QDs. When ^MUA^CdSe/ZnS QDs at concentrations greater than 5 ppm were added to the algal solution, O_2_ production rate decreased to nearly zero, and when 100 ppm ^MUA^CdSe/ZnS QDs were added, CO_2_ depletion rates significantly reduced. Because O_2_ is the end product of the photosynthesis reaction, the authors concluded that ^MUA^CdSe/ZnS QDs had detrimental effects on algal photosynthesis; however, no mechanisms for this effect were investigated.

Using fluorescence microscopy, Bouldin et al. [22] showed uptake of carboxylated CdSe/ZnS QDs by the green algae *Pseudokirchneriella subcapitata*. Algae were subjected to 96 h chronic toxicity tests using standardized methods and 96 h chronic tests following EPA protocols [23]. Algal suspensions were exposed to ^COOH  PEG^CdSe/ZnS QD suspensions ranging from 11.1 to 55.0 ppb. The algal LC50 of ^COOH  PEG^CdSe/ZnS QDs was 37.1 ppb after 96 h, which was equal to 9638 *μ*g L^−1^ of Cd and 2410 *μ*g L^−1^ of Se. Images of control and exposed algae were taken using fluorescence microscopy, and the average pixel intensity of the fluorescent images was measured. Pixels from algae exposed to 11.1 and 55.0 ppb ^COOH  PEG^CdSe/ZnS QDs had significantly increased pixel intensity (11.14 ± 17.48) when compared to controls (0.47 ± 0.13). Significant differences for pixel intensity were also measured for polygon areas of control algae (2808 ± 801) compared to algae exposed to 11.1 and 55.0 ppb ^COOH  PEG^CdSe/ZnS QDs (3332 ± 767 and 43800 ± 1522, resp.). The authors also reported physical changes in the cellular structure of algal cells, with exposed cells assuming a spherical shape when compared to the normal crescent or sickle shape of unexposed cells. The authors were also able to show the food chain transfer of ^COOH  PEG^CdSe/ZnS QDs from exposed algae to *C. dubia* by again measuring pixel intensity of fluorescent images. Unfed animals had an average pixel intensity of 9.45 ± 1.69 after 24 h. *C. dubia* fed-unexposed algae had a mean pixel intensity of 9.97 ± 0.89, and animals that fed on algae exposed to 55.0 ppb ^COOH  PEG^CdSe/ZnS QDs had a pixel intensity of 13.27 ± 0.53. No mortality was observed over the 24 h feeding period.

Gao et al. [24] investigated the effects of engineered nanomaterials on microbial-catalyzed oxidation of organic matter in freshwater sediments. The effects of octadecyl amine (ODA) ligand-coated CdSe/ZnS QDs on acetated oxidation by nitrate reducing bacteria were assessed. Using ion chromatography to measure concentrations of acetate, nitrite and nitrate, ^ODA^CdSe/ZnS QDs were shown to negatively affect acetate oxidation in sediment slurries spiked with acetate and/or nitrate when compared to control slurries (no acetate or nitrate), but the effect was not significant. The authors reported that similar results were observed for nano-Ag, while C_60_ appeared to entirely inhibit acetate oxidation.

### 2.2. Effects of QD Exposure on Aquatic Invertebrates

Ingle et al. [25] studied the absorption of commercial CdSe/ZnS QDs (Qdot 545 ITK Carboxyl Quantum Dots—Fisher Scientific, USA; Fisher part no Q21391MP-emission 541–549 nm) functionalized with carboxyl groups (^COOH^CdSe/ZnS QDs) by *Ceriodaphnia dubia* (water flea) using fluorescence microscopy. Animals were exposed for 4, 8, or 24 h to concentrations of 0, 200, 400, or 600 ppt ^COOH^CdSe/ZnS QDs. Using fluorescence microscopy, they reported that exposed animals had significantly higher pixel intensities when compared to controls within 4–8 h of exposure. At 24 h, there were no significant differences in the fluorescence intensity of animals exposed to different QD concentrations, but intensity of exposed animals remained significantly greater than that of unexposed animals. The brightest regions of exposed animals were the gut and the region of the thoracic appendages.

Lewinski et al. [26] reported similar results in *Daphnia magna*. These authors showed increased fluorescence intensity in the gut using fluorescence confocal laser scanning microscopy. *Daphnia* were exposed to CdSe/ZnS QDs coated with either poly(acrylic acid)-octylamine copolymer (PAA) conjugated to polyethylene glycol (PAA-PEG), poly(maleic anhydride-alt-1-octadecene; PMAO), or PMAO conjugated to PEG (PMAO-PEG). After uptake of all types of QDs, clearance was studied in fed and fasted *daphnia*. *Daphnia* were exposed to 7.7-nM QD concentrations for 24 h and moved to QD-free media. After 48 h, the digestive tract remained fluorescent, indicating that QDs were not completely cleared from the *Daphnia*. Scanning of the QD emission wavelength showed no spectra shift suggesting that retained QDs were not degraded. However, ICP-MS revealed that after 48 h in QD-free media, Cd levels were 15 times higher than in unfed *Daphnia* and *Daphnia* fed immediately after exposure. The total Cd levels after 48 h were also elevated compared to unexposed controls. The effect of the surface coating on QD uptake was also examined. After exposure to all types of QDs, fluorescence was primarily localized in the gut tract. However, ^PMAO^ and ^PMAO-PEG^CdSe/ZnS QDs showed significantly higher uptake after 24 h than ^PAA^ and ^PAA-PEG^CdSe/ZnS QDs. ICP-MS measurements of the total Cd in *Daphnia* exposed to ^PMAO^CdSe/ZnS QDs showed that they contained 128 ± 20 ng/*Daphnia*, while *Daphnia* exposed to ^PMAO-PEG^CdSe/ZnS QDs contained 41 ± 7 ng Cd/daphnid. The authors suggest that the ^PMAO-PEG^CdSe/ZnS QDs were not absorbed by *Daphnia* because the reduction in the negative surface charge inhibited electrostatic attraction between these QDs and biological particulates. Differences in toxicity were also observed between QDs coated with different ligands. The LC50 value for ^PMAO^CdSe/ZnS QDs was 3.1 nM, while no LC50 value could be measured for ^PMAO-PEG^CdSe/ZnS QDs at exposure concentrations up to 25.6 nM. Using DLS, ^PMAO^CdSe/ZnS QDs were also reported to form micrometer-sized aggregates in media, while aggregates of QDs with the other surface coatings could not be detected. The authors suggested that because *D. magna* can more easily retain larger particles, the increased uptake of ^PMAO^CdSe/ZnS QDs could be due to reduced particle stability and QD aggregation. These results suggest that surface coating affects QD accumulation. Because fluorescence was confined to the intestine, these results also suggest that accumulated QDs are degraded in the intestine so that Cd enters the animal after QD accumulation.

Jackson et al. [27] studied the distribution of 2.6 and 4.8 nm mercaptoundecanoic acid-capped (^MUA^CdSe) QDs in *D. magna* exposed to ^MUA^CdSe QD concentrations of 15 nmol L^−1^ for 12, 24, and 36 h, using 2D and 3D synchrotron X-ray spectroscopy. Zn and Se signals showed that the ^MUA^CdSe QDs were primarily located in the gut. 2D elemental imaging showed no evidence of  ^MUA^CdSe QD distribution within other organs. X-ray tomography showed that the ^MUA^CdSe QDs did not cross the gut epithelium. The authors suggest that possible ^MUA^CdSe QD aggregation in the gut prevented their absorption or that possibly functionalized ^MUA^CdSe QDs are large to cross cell membranes. These results also suggest that at the exposure times used, ^MUA^CdSe QDs were not degraded in the intestine. 

Together, these studies show that *Daphnia* can rapidly accumulate several types of QDs, but the resulting low toxicity and the lack of evidence for absorption of QDs by the gut epithelium suggest that the effects of acute exposure to low concentrations of QDs are probably not, by themselves lethal. However, as shown in studies described below, the type of coating, photolysis, and degradation of the coatings surrounding the QD core greatly affect toxicity and the release of Cd after accumulation. In addition, the results of studies assessing nonlethal effects of chronic exposures to QDs have not been reported. Thus, uncertainty about the effects of QDs released into the environment on these critical components of numerous ecosystems remains. 

Kim et al. [28] measured the 48 h phototoxicity of Cd and 3-mercaptopropionic-acid-(MPA-) or tri-n-octylphosphine-oxide/gum-Arabic-(GA-) coated CdSe/ZnS QDs (^MPA^CdSe/ZnS and ^MPA^CdSe/ZnS QDs) to *D. magna* under the influence of environmentally relevant UV-B light exposure. They reported that the dose-dependent toxicity of exposure to ^MPA^CdSe/ZnS QDs increased when exposure vessels were illuminated by UV-B light. They used a radiometer to measure UV-B radiation intensity at the surface of the media and test solutions and reported that it ranged from 12 to 15 *μ*W/cm^3^. The Cd acute median effective concentration (EC50) was 70.4 *μ*g L^−1^ without UV-B light and 17.3 *μ*g L^−1^ with UV-B light. ^MPA^CdSe/ZnS QDs alone showed no lethality, and ^MPA^CdSe/ZnS QDs only showed toxicity at concentrations of ~3 *μ*g L^−1^ (EC_50_ of 95.9 *μ*g L^−1^). The application of UV-B light to *D. magna* exposed to ^MPA^CdSe/ZnS QDs caused the EC_50_ to significantly decrease to 58.5 *μ*g L^−1^, but no toxicity of ^MPA^CdSe/ZnS QDs was observed even in conjunction with UV-B light. These results suggest that under environmentally relevant conditions, the impact of QDs will be dependent on their surface characteristics. The authors also compared the release of Cd from both QD types under different lighting conditions. After 2 d QD suspensions were passed through a filtration membrane and the Cd in the supernatant measured using graphite-furnace AAS. 500 *μ*g L^−1^ ^GA^CdSe/ZnS QDs incubated for 2 d in dark conditions, white light without UV-B light, and white light with UV-B light resulted in Cd concentrations of 0.196, 0.885 and 5.310 *μ*g L^−1^, respectively. Under the same conditions, Cd concentrations in suspensions of  ^MPA^CdSe/ZnS QDs were 0, 245.5, and 242.7 *μ*g L^−1^, respectively. These results suggest that Cd release from QDs is highly dependent on exposure to light and that release is affected by the wavelength and intensity of the light, but that photodegradation of QDs is also highly influenced by the outer QD coating. The authors also reported that ^GA^CdSe/ZnS QDs also were able to generate significantly increased levels of ROS as exposure concentration was increased. Using qPCR, mRNA expression levels of four Cd sensitive genes; *opsin BCRH2 (*OPS), vitellogenin (VTG), **α*-esterase* (EST), and *hemoglobin *(HEM) were measured in *Daphnia* exposed to QDs and Cd. Exposure to ^GA^CdSe/ZnS QDs resulted in increases in the expression of HEM that was not affected by dose or UV-B light. In the absence of UV-B light, ^MPA^CdSe/ZnS QD exposure produced no changes in mRNA expression. Under UV-B light exposure, VTG was significantly downregulated and decreased in a dose-dependent manner. Cd exposure significantly increased levels of OPS and VTG. Addition of UV-B caused a dose-dependent increase in HEM and VTG, but increases in EST and OPS were not dose dependent. These results suggest that QD exposure alters gene expression, in a highly gene-specific manner, that generalizations about QD effects on gene expression will be difficult and that the outer coating of the particles has profound effects on these interactions. 

The results of a similar set of experiments were reported by Lee et al. [29]. They used CdSe/ZnSe QDs coated with either mercaptopropionic acid (MPA) or gum Arabic/tri-n-octylphosphine oxide (GA/TOPO) and exposed *D. magna* in the dark, under white fluorescent light, UV-B light or sunlight. Using dynamic light scattering (DLS), ^GA/TOPO^ and ^MPA^CdSe/ZnSe QDs had hydrodynamic sizes of 65.9 and 4.5 nm, respectively. 48 h toxicity testing under standard laboratory lighting conditions revealed that ^MPA^CdSe/ZnSe QDs were not toxic to *Daphnia* at concentrations as high as 2500 *μ*g L^−1^. However, ^GA/TOPO^CdSe/ZnSe QDs had a 48 h EC50 value of 67.4 *μ*g L^−1^. The authors determined that observed toxicity was not due to the coating and reported that exposure to GA alone resulted in no toxicity at 48 h. For comparison, *Daphnia* exposed to CdCl_2_ had an EC50 of 34.1 *μ*g L^−1^. Lighting conditions were shown to have effects on the toxicity of the QDs. Natural sunlight caused the greatest increase in toxicity with 48-h EC50 values for ^GA/TOPO^ and ^MPA^CdSe/ZnSe QDs increasing 6-fold. ^GA/TOPO^CdSe/ZnSe QDs were still most toxic to *Daphnia*. CdCl_2_ EC50 values increased 18-fold under sunlight exposure. From least to most toxic, the order of lighting effects on *Daphnia* exposed to QDs toxicity was dark, white fluorescent light, UV-B light, and sunlight. The authors also studied the changes in physiochemical characteristics of  ^GA/TOPO^ and ^MPA^CdSe/ZnSe QDs in *Daphnia* after exposure to UV-B light. After exposure, spectrofluorometry showed the emission peak at 577 nm of the ^GA/TOPO^CdSe/ZnSe QDs was not present. Instead, there was a blue shift emission peak at 560 nm and a red shift emission peak at 620 nm. The authors suggest that the blue shift is indicative of a decrease in the core diameter of the QD by about 0.7 nm that could be the result of QD degradation within *Daphnia*. Conversely, the authors suggest a red shift could be caused by QD aggregation or by substitution of QD ligands by biomolecules in the *Daphnia* digestive tract.

Pace et al. [30] also studied the influence of QD size and coating on acute toxicity to *D. magna*. 48 h acute toxicity tests according to U.S. EPA Standard Test Protocol with a mortality endpoint were used [31]. CdSe/ZnS QDs (core diameter 2 and 5 nm) coated with either polyethylene oxide (POE) or MUA were used. To separate dissolved metals from whole and fragmented nanoparticles, 3,000 Dalton filtration samples were collected at 0 and 48 h and subjected to ICP-AES analysis. This showed that ^MUA^ and ^POE^CdSe/ZnS QDs produced negligible amounts of dissolved Cd and no blue shift in emission wavelength at 0 h. However, the fluorescence intensity of both the 2 and 5 nm diameter ^MUA^CdSe/ZnS QDs decreased by 80% after 48 h, while the fluorescence intensity for ^POE^CdSe/ZnS QDs remained stable. After 48 h, 61% of the Cd and 86% of the Zn from the 2 nm diameter ^MUA^CdSe/ZnS QDs were in the dissolved form. Similarly, 33% of the Cd and 14% of the Zn from the 5 nm diameter ^MUA^CdSe/ZnS QDs had dissolved. However, no significant blue shift in emission wavelength was observed showing that the size of the QDs had not changed, thus suggesting that Cd was not being released from the core. The authors suggested possible metal impurities, but analysis of the water from which the QDs were removed by ultrafiltration immediately after suspension contained no detectable Cd. The authors suggest that excess Cd was bound to the organic ligand during the synthesis process and was not fully washed away. Exposure to PEO ligands at concentrations as high as 200 mg L^−1^ (twice the PEO concentration expected in the highest QD exposure concentration) produced no *daphnia* mortality, but exposure to ^PEO^CdSe/ZnS QDs showed acute toxicity without particle dissolution. In contrast, exposure to MUA ligands at a concentration of 10 mg L^−1^ (higher than expected MUA concentration in QD exposures) caused significant mortality. Consequently, ^MUA^CdSe/ZnS QDs were significantly more toxic than ^POE^CdSe/ZnS QDs. Overall, 5 nm MUA-coated QDs were found to be more toxic than the 2 nm QDs, probably because of the increased release of dissolved metals (both on particle number and mass concentration basis) or because they contained a larger amount of MUA. The authors also reported that all intact QDs tested had lower toxicities than dissolved Cd indicating that Cd from intact QDs might not be bioavailable to *Daphnia* under the exposure conditions used. However. these results suggest that acute toxicity was the result of exposure to MUA and not to QDs or Cd released from them. 

Gagné et al. [32] studied the effects of cadmium sulfate (CdSO_4_) and CdTe cores on the freshwater mussel, *Elliptio complanata*. The CdTe QDs were not coated, nor were their surfaces altered. Mussels were exposed to CdTe QD concentrations of 0, 1.6, 4, and 8 mg L^−1^ for 24 h at 15°C. Markers of immunocompetence, oxidative stress, and genotoxicity were examined. No mussel fatalities were reported after a 24 h exposure. The authors reported that particle aggregation was observed at moderate and high exposure concentration levels, and CdTe QD dissolution was also reported, with 1.2 mg L^−1^ Cd existing in the dissolved phase in suspension containing an initial concentration of 8 mg L^−1^ CdTe QDs. After exposure, CdTe QD concentrations was measured in digestive gland and gill tissue homogenates, and the bulk of the CdTe QDs were found in the digestive glands. No CdTe QDs were detected in the gills. The authors next examined the effects of exposure to the CdSe QDs on the immune system by measuring the viability and phagocytic activity of hemocytes collected from the posterior adductor sinus. The authors reported that initial phagocytic activity and cell viability were about 15% lower than those observed in other marine bivalves. Hemocyte phagocytosis was measured by incubated cells with 2 *μ*m red latex FluoSpheres at a cell/bead ratio of 1 : 30. After 18 h incubations in the dark, active hemocytes were measured using cytometry. They reported that phagocytosis of fluorescent beads showed a 3.9-fold inhibition at a QD concentration of 4 mg L^−1^. Exposure of mussels to CdSO_4_ reduced phagocytic activity by 1.3-fold. The authors were also able to show an increased capacity of the hemocytes to lyse mammalian K-562 cells at CdTe QD concentrations greater than 5.6 mg L^−1^. Oxidative stress (OS) was measured in the gills and digestive glands by examining lipid peroxidation levels. Exposure to 5.6 mg L^−1^ CdTe QDs induced a 1.4-fold increase in OS in the gills. By comparison, exposure to 0.5 mg L^−1^ CdSO_4_ caused a 1.6-fold increase in the gills. However, exposure to CdTe QDs reduced OS in the digestive glands. For example, exposure to 8 mg L^−1^ CdTe QDs induced a 2-fold reduction in digestive gland OS, while 0.5 mg L^−1^ CdSO_4_ reduced lipid peroxidation levels 1.3-fold. Using Olive's alkaline DNA precipitation assay, DNA strand breaks were shown to significantly decrease in the gills at CdTe QD concentrations of 1.6 and 4 mg L^−1^, and in the digestive glands after exposure to 4 and 8 mg L^−1^ CdTe QDs indicating a loss of DNA repair activity, but returned to control values at the highest CdTe QD concentration tested. In comparison, treatment with CdSO_4_ had no effect on DNA strand breaks. 

The same group studied the effect of CdTe QDs on cadmium bioaccumulation and metallothionein (MT) production in *E. complanata* [33]. Using a series of filtration membranes (pore sizes: 450 nm, 100 nm, 50 nm, 25 nm, and 1 kDa), the authors reported that after 24 h in aquarium water, 80% of Cd from CdTe QDs was in aggregate form, while 14% was in the dissolved form. Atomic absorption spectrometry (AAS) of acid-digested tissues showed that mussels exposed to CdTe QDs for 24 h at 15°C and allowed to depurate in clean aquarium water for 24 h had Cd in their gills, digestive gland, and gonad tissues. Gills contained the most Cd, suggesting CdTe QDs were readily absorbed by the gills. Exposure to CdTe QD concentrations of 1.6, 4 and 8 *μ*g L^−1^ yielded Cd levels in the gills of 15.8, 13.5, and 16.2 *μ*g Cd g^−1^ dry tissue, respectively. MT levels decreased significantly in the gills at the threshold CdTe QD concentration of 5.6 mg L^−1^, a 1.9-fold reduction compared to unexposed animals. 0.3 mg L^−1^ CdSO_4_ also reduced MT levels in the gills. However, MT levels significantly increased in the digestive gland at a threshold CdTe QD concentration of 1.6 mg L^−1^ with a 1.8-fold induction at 4 mg L^−1^ CdTe QDs. Exposure to the largest concentrations caused a return to preexposure MT levels. Exposure to a concentration of 4 mg L^−1^ QDs also caused a significant increase in MT levels in the gonad. However, this measurement technique did not allow these investigators to distinguish between Cd from degraded and intact CdTe QDs.

### 2.3. Effects of QD Exposure on Aquatic Vertebrates

Gagné et al. [34] studied the effects of CdTe capped with carboxylated Cd/S QDs (^COOH-Cd/S^CdTe QDs) on the immune system of *Oncorhynchus mykiss*. Fish were exposed to ^COOH-Cd/S^CdTe QDs concentrations of 1, 2, and 6 *μ*g L^−1^ or CdSO_4_ concentrations of 0.6, 1, and 2 *μ*g L^−1^ for 96 h at 15°C. Cd concentrations were first measured in the aquarium water with nearly all Cd from CdSO_4_ in the dissolved phase, but only 0.4% of the Cd from ^COOH-Cd/S^CdTe QDs was in solution. The head kidney was extracted so that the ratio of live/dead leukocytes and macrophage phagocytic activity could be measured. Leukocyte concentrations decreased ~1.3-fold in animals exposed to the lowest exposure concentrations of ^COOH-Cd/S^CdTe QDs or CdSO_4_. Exposure to 2 *μ*g L^−1^ Cd resulted in a decrease in leukocyte viability from 76% to 68%, but cell viability increased in fish exposed to 6 *μ*g L^−1^ ^COOH-Cd/S^CdTe QDs. Phagocytic activity of phagocytes was evaluated by exposing cell suspensions to fluorescent latex beads at a cell/bead ratio 1 : 100. Resting phagocyte (phagocytes having engulfed ≥ 1 fluorescent beads) activity was reduced by exposure to 1 and 2 *μ*g L^−1^ ^COOH-Cd/S^CdTe QD and unaffected by exposure to CdSO_4_. Active phagocytes (phagocytes having engulfed ≥ 3 fluorescent beads) were affected similarly although activity was also reduced when exposed to only 1 *μ*g L^−1^ CdSO_4_. Liver tissue was harvested from these animals, and gene expression was assessed using a DNA microarray that contained 207 stress-related genes. 36 of the 207 genes showed at least a 2-fold response (increase or decrease) when fish were exposed to 1 *μ*g L^−1^ ^COOH-Cd/S^CdTe QD or CdSO_4_. The expression of 25 genes was affected only by exposure to ^COOH-Cd/S^CdTe QDs. 10 genes were highly affected, showing a 4-fold change in expression. In total, 13 genes were downregulated and 15 were upregulated by exposure to ^COOH-Cd/S^CdTe QDs. 24 of these genes were involved in at least one immune response endpoint. In contrast, expression of 9 genes was affected only by CdSO_4_ exposure; three genes were downregulated, and 6 genes were upregulated. 8 of these genes were involved in immune response endpoints. It is interesting to note that the authors reported that vitellogenin (VTG) levels increased when fish were exposed to ^COOH-Cd/S^CdTe QDs, but not when exposed to CdSO_4_. However, a follow-up *in vitro* study exposing trout hepatocytes to ^COOH-Cd/S^CdTe QDs or CdSO_4_ showed the VTG expression was not affected by either. The authors suggest that VTG induction was a systemic affect and not an induction of gene expression at the cellular level. The expression of three genes, metallothionein 1A (MT1A), cytochrome P450 2K1 (CYP2K1 and 2K4), and retinol-binding protein (RBP4), was affected by both ^COOH-Cd/S^CdTe QD and dissolved CdSO_4_. The authors suggest that these different responses to dissolved Cd and intact ^COOH-Cd/S^CdTe QDs indicate that it is likely that Cd leaching from ^COOH-Cd/S^CdTe QDs was not responsible for the observed effects. These data suggest that mechanisms by which QDs alter gene expression are different from those of dissolved Cd, providing strong evidence that further research into the molecular and chemical mechanisms by which QDs interact with cell components is needed if we are to understand QD toxicity. Studying the effects of the constituent metals is not sufficient to explain these observations.

King-Heiden et al. [35] studied the nanotoxicity of CdSe/ZnS QDs functionalized with poly-L-lysine (PLL) or poly(ethylene glycol) (PEG) terminated with methoxy (OCH_3_), carboxylate (COO–), or anime (NH_2_) groups on zebrafish embryos. DLS and electrophoretic light scattering were used to assess the stability of all types of QDs in ddH_2_O and zebrafish embryo media (58 mM CaCl_2_, 0.7 mM KCl, 0.4 mM MgSO_4_·7H_2_O, 0.6 mM Ca(NO_3_)_2_·4H_2_O, 0.5 mM HEPES, pH 7, and ionic strength 0.18) for 24 h at 28°C. All QD hydrodynamic diameters were at the values expected of single particles in ddH_2_O except for ^PLL^CdSe/ZnS QDs, which were larger than expected, indicating possible aggregation. The same responses were observed in the embryo medium except that the ^PEG350-OCH3^CdSe/ZnS and ^PLL^CdSe/ZnS QDs aggregated into particles that had hydrodynamic diameters 85 and 42 times larger than expected of individual particles. The *ζ*-potentials of the aggregated QDs were positive, while the stable suspensions had slightly negative *ζ*-potentials. UV-visible absorbance spectra of all QD suspensions showed no blue shift in QD emission wavelengths indicating no change in core diameters of any of the tested QDs. ICP-OES showed that less than 5% of the total Cd was released from ^PEG350-OCH3^CdSe/ZnS, ^PEG5000-COO*- *^CdSe/ZnS, and ^PEG5000-OCH3^CdSe/ZnS QDs. However, ^PLL^ and ^PEG5000-NH2^CdSe/ZnS QDs showed Cd release of 19% and 14%, respectively. Embryos were independently exposed to all types of QDs at concentrations ranging from 0 to 1600 *μ*M (0.2–200 *μ*M Cd equivalents) for 120 h beginning at 4–6 h after afertilization, and toxic end points were measured. The authors reported that ^PEG350-OCH3^CdSe/ZnS QDs showed low toxicity, while ^PLL^QDs were significantly more toxic than all forms of ^PEG^QDs. With the exception of the ^PEG5000-NH2^CdSe/ZnS QDs, exposure to all types of QDs was associated with greater increases in zebrafish embryo mortality than the equivalent concentrations of CdCl_2_. Because none of the QDs used in the study released significant amounts of Cd, the authors suggest that the observed effects were probably not due to direct Cd exposure. Further characterization of the differential effects of Cd and QDs was done by exposing embryos to a single 20 *μ*M dose of CdCl_2_ (Cd) or a 2 *μ*M Cd-equivalent dose of QDs. Several differences in sublethal effects were reported. Cd exposure is known to cause dose- and age-dependent end points including reduced growth, bent spine, and edema of the ocular, pericardial, submandibular, and yolk sac [36, 37]. Embryos exposed to all types of QDs except ^PLL^ and ^PEG350-OCH3^CdSe/ZnS QDs exhibited the same malformations, but also exhibited necrosis, yolk sac malformation, and a malformed tail. However, embryos exposed to ^PLL^ and ^PEG350-OCH3^CdSe/ZnS QDs showed no malformities. Using graphite furnace atomic-absorption spectrophotometry (GFAA), the authors also reported that the size of QD aggregates affected the total Cd body burden and toxicity. QDs that formed larger aggregates also showed reduced uptake. Zebrafish larvae exposed to over 2 *μ*M Cd-eq of ^PEG5000^CdSe/ZnS QDs and 20 *μ*M Cd-eq of ^PLL^CdSe/ZnS QDs accumulated a considerable Cd body burden. Zebrafish exposed to ^PLL^CdSe/ZnS and ^PEG350-OCH3^CdSe/ZnS QDs had lower Cd body burdens compared to fish exposed to other types of QDs or CdCl_2_. Measurements of metallothionein (MT) expression showed that embryos exposed to all types of QDs had a dose-dependent increase in MT expression, leading the authors to suggest partial QD breakdown *in vivo *as the mechanism for this effect. MT expression was affected by CdCl_2_ exposures in a dose-dependent manner. Interestingly, exposure to QDs that had an increased tendency to aggregate was correlated with increased MT levels and reduced toxicity. The authors also showed that PLL was itself toxic, so ^PLL^QDs were more toxic than other types of QDs tested. This explained the high toxicity, but low Cd body burden associated with exposure to ^PLL^QDs.

Leigh et al. [38] measured the uptake and depuration of commercial CdSe/ZnS QDs expressing surface carboxyl groups in the fathead minnow, *Pimephales promelas*. Fish were exposed to 0.1, 0.5, 1, or 2 nM concentrations of ^COOH^CdSe/ZnS QDs for 5, 24, or 72 h, and dose- and time-dependent bioaccumulation was characterized. Fluorometry was used to measure QD concentrations accumulated in intestinal tract of the fish. A dose-dependent accumulation was observed in fish exposed to ^COOH^CdSe/ZnS QDs with those exposed to 0.5, 1, or 2 nM concentrations accumulating gut QD concentrations of ~17, 34, and 32 nM, respectively. No measureable concentration was detected in fish exposed to 0.1 nM QDs. Fish exposed to 2 nM ^COOH^CdSe/ZnS QDs for 24 h were shown to be capable of expelling accumulated QDs when placed in clean water for 24 h. Fish exposed to 2 nM ^COOH^CdSe/ZnS QDs for 5, 24, or 72 h accumulated ~15, 34, and 13 nM of QDs in their gut, respectively. The authors suggest that a degradation of accumulated QDs could be responsible for the reduction in gut QD concentration observed after 72 h exposure. The authors followed by measuring ^COOH^CdSe/ZnS QD concentrations in the suspension to which fish were exposed over 72 h. It was shown that suspensions with fish caused significantly greater decreases in ^COOH^CdSe/ZnS QD concentration compared to suspensions without fish. The authors suggest that fish/QD interactions are responsible for the loss of detectable QDs, and that QD degradation may be responsible for the observed reductions in detectable QDs.

## 3. Conclusions

The literature reviewed here suggests that QD physiochemical and environmental factors must be taken into account together when studying the environmental fate of QDs in aquatic environments. Because QDs can have multiple configurations (i.e., core/shell metals, size, and surface chemistry), it is important that each configuration be examined separately in environmental studies. Furthermore, each QD must be studied under different environmental conditions (i.e., acidity/alkalinity, salt/fresh water, NOM composition, temperature, contaminants, etc) to provide truly useful information about how QDs will behave in aquatic environments. This review suggests that the toxicity of QDs to aquatic organisms appears to initially rely heavily on the surface ligands of the QDs and be affected by exposure to oxidative environments; it also suggests that the primary mechanism of QD toxicity is generation of ROS as the QD surface chemistry reacts with its surrounding environment. There is very little reported data relevant to understanding how QDs released into the environment may interact with aquatic organisms and ecosystems. For example, there is very little information about how QDs may partition in environmentally relevant substrates, especially sediment and water. More information about potential trophic interactions and accumulation is much needed as is more information about the mechanisms by which QDs may be degraded and excreted by organisms. There is also a paucity of studies about the effects of chronic exposures to QDs. Although work in this field is progressing rapidly, it is apparent that studies regarding QD environmental fate are not keeping up with the increasing production and usage of QDs and that our knowledge of the interactions of these particles with living organisms must be greatly expanded.
